# Supplementary material for: Impacts of the COVID-19 pandemic on food prices: Evidence from storable and perishable commodities in India
Source: PLoS One. 2022 Mar 3;17(3):e0264355. doi: 10.1371/journal.pone.0264355 (PMC8893685; doi:10.1371/journal.pone.0264355)
Supplement: S1 Table — (DOCX) [file pone.0264355.s001.docx]

**S1 Table. The effects of the COVID-19 pandemic on food prices in Asian economies: Results from previous studies.**

| Country | Data used | Storable foods | Perishable foods | Effects of COVID-19 on price | Source |
| --- | --- | --- | --- | --- | --- |
| Bangladesh | - Farm price (survey) |  | - Vegetables | - Dropped more than 50% | Alam and Khatun [39] |
| China (151 markets) | - Wholesale prices (time series) | - Rice - Wheat flour |  | - No significant changes - No significant changes | Ruan, Cai, and Jin [17] |
|  |  |  | - Chinese cabbage | - Increased significantly (11-65%) |  |
| China (Shandong, Beijing, and Hubei) | - Wholesale prices (time series) |  | - Chinese cabbage | - Increased significantly | Yu et al. [18] |
| China | - Wholesale prices (time series) |  | - Fresh fruits and vegetables | - Increased by about 10% | Çakır, Li, and Yang [19] |
| India (urban food markets) | - Wholesale and retail prices (time series) | - Rice - Atta - Edible oils - Pulses |  | - 0.98% increased - 0.54% increased - 3.5% increased - 6% increased | Narayanan and Saha [13] |
|  |  |  | - Potato - Tomato | - 15% increased - 28% increased |  |
| India (Maharashtra, Jharkhand, and Meghalaya) | - Wholesale and retail prices (time series) | - Rice |  | - Increased | Imai, Kaicker, and Gaiha [9] |
|  |  |  | - Onion - Potato - Tomato | - Increased - Increased - Increased |  |
| India (four districts in Haryana) | - Farm gate prices (phone interview) | - Wheat |  | - Negligible effect | Ceballos, Kannan, and Kramer [14] |
|  |  |  | - Tomato | - Fell steeply |  |
| India (Jammu and Kashmir) | - Wholesale prices (time series) |  | - Onion - Vegetables - Fruits | - Increased - Declined by 19% - Declined by 11-39% | Ali and Khan [15] |
| India (Jharkhand, Assam, Andhra Pradesh, and Karnataka) | - Producer prices (phone survey) |  | - Vegetables | - 80% of farmers reported a decline in prices | Harris et al. [16] |
| Myanmar | - Millers’ prices (face-to-face survey) | - Local rice - Exported rice |  | - No significant changes - Increased significantly | IFPRI [12] |

Source: Authors’ compilation from various sources.
