# Supplementary material for: Impacts of the COVID-19 pandemic on food prices: Evidence from storable and perishable commodities in India
Source: PLoS One. 2022 Mar 3;17(3):e0264355. doi: 10.1371/journal.pone.0264355 (PMC8893685; doi:10.1371/journal.pone.0264355)
Supplement: Attachment — Submitted filename: PONE_response_letter_R1_v2.0.docx [file pone.0264355.s002.docx]

PONE-D-21-29286

**Impacts of the COVID-19 pandemic on food prices: Evidence from storable and perishable commodities in India**

Dear Dr. Bairagi,

Thank you for submitting your manuscript to PLOS ONE. After careful consideration, we feel that it has merit but does not fully meet PLOS ONE’s publication criteria as it currently stands. Therefore, we invite you to submit a revised version of the manuscript that addresses the points raised during the review process.

Please submit your revised manuscript by 11.11.2021. If you will need more time than this to complete your revisions, please reply to this message or contact the journal office at [plosone@plos.org](mailto:plosone@plos.org). When you're ready to submit your revision, log on to https://www.editorialmanager.com/pone/ and select the 'Submissions Needing Revision' folder to locate your manuscript file.

Please include the following items when submitting your revised manuscript:

- A rebuttal letter that responds to each point raised by the academic editor and reviewer(s). You should upload this letter as a separate file labeled 'Response to Reviewers'.
- A marked-up copy of your manuscript that highlights changes made to the original version. You should upload this as a separate file labeled 'Revised Manuscript with Track Changes'.
- An unmarked version of your revised paper without tracked changes. You should upload this as a separate file labeled 'Manuscript'.

If you would like to make changes to your financial disclosure, please include your updated statement in your cover letter. Guidelines for resubmitting your figure files are available below the reviewer comments at the end of this letter.

We look forward to receiving your revised manuscript.

Kind regards,

László Vasa, PhD
Academic Editor
PLOS ONE

*Dear Dr. Vasa and reviewers,*

*We are grateful for the constructive feedback and the high-quality referee reports we have received. We are pleased to see that our manuscript has been enthusiastically received by all of the four reviewers and their comments helped us to sharpen our manuscript even further. We have formulated our point-to-point responses to the comments in colored text.*
**Comments to the Author**

1. Is the manuscript technically sound, and do the data support the conclusions?

The manuscript must describe a technically sound piece of scientific research with data that supports the conclusions. Experiments must have been conducted rigorously, with appropriate controls, replication, and sample sizes. The conclusions must be drawn appropriately based on the data presented.

Reviewer #1: Yes

Reviewer #2: Partly

Reviewer #3: Yes

Reviewer #4: Partly

2. Has the statistical analysis been performed appropriately and rigorously?

Reviewer #1: Yes

Reviewer #2: N/A

Reviewer #3: Yes

Reviewer #4: I Don't Know

3. Have the authors made all data underlying the findings in their manuscript fully available?

The [PLOS Data policy](http://www.plosone.org/static/policies.action#sharing) requires authors to make all data underlying the findings described in their manuscript fully available without restriction, with rare exception (please refer to the Data Availability Statement in the manuscript PDF file). The data should be provided as part of the manuscript or its supporting information, or deposited to a public repository. For example, in addition to summary statistics, the data points behind means, medians and variance measures should be available. If there are restrictions on publicly sharing data—e.g. participant privacy or use of data from a third party—those must be specified.

Reviewer #1: No

Reviewer #2: Yes

Reviewer #3: Yes

Reviewer #4: No

4. Is the manuscript presented in an intelligible fashion and written in standard English?

PLOS ONE does not copyedit accepted manuscripts, so the language in submitted articles must be clear, correct, and unambiguous. Any typographical or grammatical errors should be corrected at revision, so please note any specific errors here.

Reviewer #1: Yes

Reviewer #2: No

Reviewer #3: Yes

Reviewer #4: Yes

**5. Review Comments to the Author**

Please use the space provided to explain your answers to the questions above. You may also include additional comments for the author, including concerns about dual publication, research ethics, or publication ethics. (Please upload your review as an attachment if it exceeds 20,000 characters)

**Reviewer #1:**

I liked your paper: you chose a significant topic, and wrote in a very logical and comprehensive manner. I would only recommend to indicate at the end of the manuscript the limitations of the study (i.e. that transfers and remittances were not included in the model), or giving hints for further research possibilities.

***Response:*** *Thank you. We have added a limitation section with future research possibilities at the end of the manuscript based on your suggestion.*

**Reviewer #2:**

Dear Author,

While reading your work, I found the topic interesting but at the same time, I find some lack of proper design and limited methodology. As such, I want to draw the following issues:

***Response:*** *Thank you for the positive appreciation of our paper and the constructive feedback. We have formulated our point-to-point responses to the comments in colored text.*

1- The literature gap and the novelty of this work are not evident and clear;

***Response:*** *You are right that our contribution was unclear in the previous version. Therefore, as were suggested by two other reviewers, we have added a new literature review section in the revised version. More importantly, we have also revised the introduction section that clearly explains the literature gap and the contribution of our paper [please see pages 1-7].*

2- There is a lack of data analysis and synthesis of the results;

***Response:*** *Indeed, your point is well taken. Our objective of the study is partly motivated by the availability of data and contribution to the literature. We noted that COVID indeed fueled several studies around the world. However, many of the studies performed basic data analysis and reported the average changes of outcome variables before and after the lockdowns. Considering this, our study is much more rigorous because we have utilized a standard econometric method with recently collected quality survey data by the World Bank.*

*The World Bank collected data using telephone surveys from six states in India. The data is timely but not be comprehensive. Only limited economic indicators were collected. Based on the limited variables available in that dataset, our study is the first to estimate the effect of COVID-Pandemic on the prices of perishable and storable commodities in India. We beg to differ from our comment.*

*Finally, you can find a discussion of data in the “Survey data and descriptive statistics” section. Further, several findings drawn from regression (difference-in-differnce) analysis are presented in the “Results and Discussion” section of the paper.*

3- Methodology used is very simple and does not offer a complete picture of what really happened to the consumption of the three categories you present.

***Response:*** *This is a very insightful comment, a demand function without quantity. However, since the World Bank’s data do not have consumption data, we adopted a similar method following Ruan et al. (2021)*

*Ruan J, Cai Q, Jin S. Impact of COVID-19 and nationwide lockdowns on vegetable prices: Evidence from wholesale markets in China. Am J Agric Econ. 2021;00: 1–21. doi:10.1111/ajae.12211*

I hope you will take into consideration these comments and improve your work accordingly.
Best wishes!

*Response: Thanks for taking the time to review our paper. We also stand to make any changes should you have.*

**Reviewer #3:**

The article discusses an interesting topic given the impact of the covid crisis on the price of staple foods in developing countries. The current covid crisis has affected food security in developing countries, as a result of dysfunctions in supply chains. The authors motivated well the choice of the three foods for their study considering their share in the consumption of the local population but also India's position on the international market

The article has the potential to be published but needs to be restructured and improved.

***Response:*** *Thank you for the positive appreciation of our paper and the constructive feedback. We have formulated our point-to-point responses to the comments in colored text.*

My main recommendations are:
1. The authors could emphasize, in the introduction, the covid crisis relationship - food security and SDGs (SDG 2 - zero hunger).

***Response****: We have entirely changed the introduction section, mainly focusing on the economics of price changes (supply and demand factors). We believe the current section reads better.*

2. The introductory section is too long, I propose to break it down into two sections Introduction and Literature review.

*Response: Based on your suggestion, we have divided the introductory section into an introduction and literature review sections..*

3. In the Literature review section, the presented studies could be divided into several categories considering the complexity of the analyzed phenomenon –for example the impact of the covid crisis on supply chains, the impact of the covid crisis on population behavior (compulsive shopping, herd behavior) etc.

For this section, additional refences coul be used
a) Alam, G. M., & Khatun, M. N. (2021). Impact of COVID-19 on vegetable supply chain and food security: Empirical evidence from Bangladesh. Plos one, 16(3), e0248120.
b) Di Crosta, A., Ceccato, I., Marchetti, D., La Malva, P., Maiella, R., Cannito, L., ... & Di Domenico, A. (2021). Psychological factors and consumer behavior during the COVID-19 pandemic. PloS one, 16(8), e0256095.
c) Loxton, M., Truskett, R., Scarf, B., Sindone, L., Baldry, G., & Zhao, Y. (2020). Consumer behaviour during crises: preliminary research on how coronavirus has manifested consumer panic buying, herd mentality, changing discretionary spending and the role of the media in influencing behaviour. Journal of risk and financial management, 13(8), 166.

***Response:*** *We have rewritten the literature section in the revised manuscript. Also, we have cited the above three articles in the relevant sections.*

4. In the conclusions section, the authors must present the limits of the research (for example, the small number of products analyzed) and the future directions of research.

***Response:*** *Thank you. We have added a limitation section in the revised version. The texts read as [lines ]:*

*“Despite insightful and thought-provoking results, this research is not without limitations. Two limitations are worth noting: we considered a few essential commodities (three) and a smaller sample, particularly for estimating rice price. Future studies could use more recent data with various food items and newer estimation techniques. Also, considering the entire supply chain, identifying the factors of erratic price behavior during a crisis would provide more insights to policymakers to design an effective price stabilization policy. Finally, since India is heterogeneous in different tastes and preferences, future studies could examine consumption data (price and quantities) of more durable and non-durable food items.”*

5. The theoretical and practical implications of the study need to be extended. Please, see for example …………..Khan, S. A. R., Razzaq, A., Yu, Z., Shah, A., Sharif, A., & Janjua, L. (2021). Disruption in food supply chain and undernourishment challenges: An empirical study in the context of Asian countries. Socio-Economic Planning Sciences, 101033.

***Response:*** *Indeed, in response to your comments, we have cited the above reference. We have included the reference in the introduction section and the policy implications section. We have already mentioned the implications that we can draw from our study in the last section of the paper. However, we feel that any further extrapolation of practical implications will be beyond the results, unjust and harmful for the readers of this paper.*

6. The population was affected differently by this black swan event, the vulnerable categories having to be in the attention of the public authorities. Specific measures should be proposed and implemented by public authorities to reduce the impact of the covid crisis on vulnerable groups. Please, see for example
a) Singh, D. R., Sunuwar, D. R., Shah, S. K., Sah, L. K., Karki, K., & Sah, R. K. (2021). Food insecurity during COVID-19 pandemic: A genuine concern for people from disadvantaged community and low-income families in Province 2 of Nepal. Plos one, 16(7), e0254954.

***Response:*** *We have added this article to our paper. We are glad to inform you that we are also conducting another research focusing on the COVID-19 and food security issues in India, where citing this paper might be more appropriate.*

7. In the discussion section, the authors must present the results of similar studies that confirm or refute the conclusions of the analysis performed by them.

***Response:*** *Thank you for your comment. Many studies have performed basic data analysis and reported the average changes before and after lockdowns to analyze the pandemic's impact. World Bank collected data using telephone surveys in India. The data is timely, but not be comprehensive. Our study is the first to estimate the effect of COVID-Pandemic on the prices of perishable and storable commodities in India. In the revised paper’s “Results and Discussion” section, we have mentioned studies that we find relevant to our paper in either supporting or refuting the results of our analysis. For example, we have mentioned Narayanan and Saha (2021);* *Varshney et al. (2020);* *Imai, Kaicker and Gaiha (2020).*

1. *Narayanan S, Saha S. Urban food markets and the COVID-19 lockdown in India. Glob Food Sec. 2021;29: 100515. doi:10.1016/j.gfs.2021.100515.*
2. *Varshney D, Roy D, Meenakshi J V. Impact of COVID-19 on agricultural markets: assessing the roles of commodity characteristics, disease caseload and market reforms. Indian Econ Rev. 2020;55: 83–103. doi:10.1007/s41775-020-00095-1*
3. *Imai, Katsushi. S., Kaicker, N. and Gaiha, Raghav. (2020) The Covid-19 impact on food prices in India. GDI Working Paper 2020-051. Manchester: The University of Manchester*

8. Certain phrases need to be reworded (certain words are repeated or the authors have not found the best wording For example line 325-326 ”Therefore, governments in India could take necessary actions such as distributing staple foods from its public food distribution system”

***Response:*** *We believe that the revised version reads better. For instance, we have changed the above sentence to: “Therefore, the government of India should take necessary policy actions to stabilize food prices, such as supplying more staple foods from the public food distribution system into the market during a crisis”.*

**Reviewer #4:**

Quality of paper is average in its current state. Do brainstorming, read more and conceptualise a clear idea. Indians do not only eat aata, Onion and Rice. India being the largest producer of food grains and with large storages of food grains, there was no scarcity of food.
Refer: https://www.indiatoday.in/india/story/in-6-years-over-40-000-tonnes-of-food-grains-damaged-in-fci-godowns-1696650-2020-07-03
https://www.indexmundi.com/agriculture/?country=in&commodity=wheat&graph=production
Wheat and Rice is not the only consumable by Indians. Indians do consume Pulses, oil seeds, Fruits, vegetables, Wheat, Jawar, Bajra and other types of millets.
Though supply chain was a challenge, it did not have burden on supply side. Hording by end users, wholesalers and retailers had impact. Government policies had positive impact.
While talking about ‘walmart, flipkart’ and ‘, smallholders opted for local retail markets’ you missed avenue supermarkets and reliance retail.
Structure and Flow of paper should be: Introduction, Structured Literature Review, Research Questions / Hypothesis, Research Methodology, Analysis of Data and Discussion, Findings and Conclusion. Research questions must arise after Literature review.
How this study of ‘COVID-19 pandemic on food prices: 2 Evidence from storable and perishable commodities in India’ case will be of interest for international readers of the journal?

***Response:*** *Thank you for your comment. Indeed, we note that Indians eat all kinds of perishable and storable commodities. Our objective of the study is partly motivated by the availability of data and contribution to the literature.*

*We noted that COVID indeed fueled several studies around the world. However, much of the studies performed basic data analysis and reported means before and after lockdowns. World Bank collected data using telephone surveys in India. The data is timely, but not be comprehensive. Our study is the first to estimate the effect of COVID-Pandemic on the prices of perishable and storable commodities in India.*

*In this study, nowhere have we mentioned the scarcity of food. That is not our intention and goal of the paper.*

*Yes, you are right that hoarding and market manipulation may be in play. However, we do not observe that in our data set. We are just telling the story of why we find significant changes in prices of commodities (World Bank collected dataset). We provide justification of why the results hold.*

*We agree that government policy may have prevented people from going hungry and suppliers using rent-seeking behavior to extract maximum profits. But, we cannot support this claim through our data. The World Bank data did not collect government intervention and types of interventions that prevented prices from rising or falling. Indeed, we refer to Varshney et al. (2020) paper that clarifies government intervention may have eased suffering along the supply chain.*

*We have followed the standard format of published papers in PLOS one. We have added another missing component of the Literature Review to the sequence that you have mentioned above.*

*We describe the data in “Survey data and descriptive statistics” section of the paper. Further, use regression analysis to analyze the data and also present the findings in the “Results and Discussion” section of the paper*

There is no useful research output in this paper. There are generalised statements and parameters.
I conclude that paper do not contribute significantly to the body of knowledge or create any significant new knowledge to justify.

***Response:*** *Thank you. We are glad to see that the other three reviewers find our paper very interesting. I hope after reading our revised version, you will change your opinion and be satisfied.*

Paper must be properly formatted and rewritten.

***Response:*** *We have properly formatted and rewritten based on the other three reviewers’ comments and the Journal’s guidelines.*

Please verify all calculations and eliminate errors if any.

*Response: As you suggested, we have carefully verified all calculations in the revised version. Thank you.*

I suggest major revision.

Implement changes and resubmit paper.

***Response:*** *We have thoroughly made changes based on the comments of the three other reviewers.*

Best Wishes.

***Response:*** *Thank you.*
