# Supplementary material for: Impacts of the COVID-19 pandemic on food prices: Evidence from storable and perishable commodities in India
Source: PLoS One. 2022 Mar 3;17(3):e0264355. doi: 10.1371/journal.pone.0264355 (PMC8893685; doi:10.1371/journal.pone.0264355)
Supplement: Attachment — Submitted filename: PONE_response_to_reviewer4_R2_v1.0.docx [file pone.0264355.s003.docx]

**PONE-D-21-29286R1**

**Title: Impacts of the COVID-19 pandemic on food prices: Evidence from storable and perishable commodities in India**

**Response to Reviewer 4**

**Comment:** I appreciate efforts taken by authors to revise paper as per comments of several reviewers. Still please try to implement few further changes. Structure and Flow of paper should be: Introduction, Structured Literature Review, Research Questions / Hypothesis, Research Methodology, Analysis of Data and Discussion, Findings and Conclusion. Research questions must arise after Literature review.

***Response: Done.*** *We have followed the paper structure that is compatible with economic analysis papers in PLOSONE*

**Comment:** Please verify all calculations and eliminate errors if any.

***Response:*** *In response to your comments, we have checked our estimates, and all are in good shape.*

**Comment:** I ask you to refer following articles for making literature review strong:
[https://doi.org/10.1371/journal.pone.0256921](https://urldefense.com/v3/__https:/doi.org/10.1371/journal.pone.0256921__;!!IKRxdwAv5BmarQ!I1uLH883Yu2Uw9R3-geTpyMk39qpxNBJlc6h177PMWmGTAp1kloSYmLGAc8rB_vjFDc$) , [https://doi.org/10.1504/ijenm.2021.118057](https://urldefense.com/v3/__https:/doi.org/10.1504/ijenm.2021.118057__;!!IKRxdwAv5BmarQ!I1uLH883Yu2Uw9R3-geTpyMk39qpxNBJlc6h177PMWmGTAp1kloSYmLGAc8r6QHQTNk$) , [https://doi.org/10.1371/journal.pone.0254954](https://urldefense.com/v3/__https:/doi.org/10.1371/journal.pone.0254954__;!!IKRxdwAv5BmarQ!I1uLH883Yu2Uw9R3-geTpyMk39qpxNBJlc6h177PMWmGTAp1kloSYmLGAc8rIdZ54Bw$)

***Response:*** *We have added these articles in the literature review section of the revised paper. However, we did not include Huang’s study in our paper. This paper is focused on West Virginia, USA, which is outside our focal areas of Asia.*

**Comment:** Implement changes and resubmit paper.

***Response:*** Done
